# Chromosomal Aberration t(14;17)(q32;q21) Simultaneously Activates HOXB5 and miR10a in Triple-Hit B-Cell Lymphoma

**DOI:** 10.3390/biomedicines11061758

**Published:** 2023-06-19

**Authors:** Stefan Nagel, Claudia Pommerenke, Corinna Meyer, Maren Kaufmann, Roderick A. F. MacLeod

**Affiliations:** Department of Human and Animal Cell Lines, Leibniz-Institute DSMZ—German Collection of Microorganisms and Cell Cultures, Inhoffenstr. 7B, 38124 Braunschweig, Germany

**Keywords:** Hodgkin lymphoma, homeodomain, HOX, KCNJ12, miR17, miR196a

## Abstract

*BCL2*, *BCL6* and *MYC* are major oncogenes in B-cell lymphoma. Their aberrant activation frequently occurs via chromosomal translocations which juxtapose light or heavy chain immunoglobulin (IG) genes to *BCL2* and *MYC* or fuse diverse partner genes with *BCL6*. So-called double-hit lymphomas usually carry *BCL2* and *MYC* rearrangements, while triple-hit lymphomas additionally bear *BCL6*-fusions. All these translocations are of diagnostic relevance and usually denote poor prognosis. Here, we genomically characterized classic follicular lymphoma (FL) cell line SC-1, thereby identifying t(14;18)(q32;q21) juxtaposing *IGH* and *BCL2*, t(8;14)(q24;q32) juxtaposing *IGH* and *MYC*, and t(3;3)(q25;q27) fusing *MBNL1* to *BCL6*. In addition, we found that SC-1 carries a novel chromosomal rearrangement, t(14;17)(q32;q21), which, though present at establishment, has remained unreported until now. We further show that t(14;17)(q32;q21) juxtaposes *IGH* with the HOXB gene cluster at 17q21 and affect the oncogenic activation of both homeobox gene *HOXB5* and neighboring micro-RNA gene *miR10a*. Moreover, we detected aberrant overexpression of *HOXB5* in subsets of Burkitt lymphoma, FL, and multiple myeloma patients, confirming the clinical relevance of its deregulation. In SC-1, *HOXB5* activation was additionally supported by co-expression of hematopoietic stem cell factor ZNF521, indicating an aberrant impact in cell differentiation. Functional investigations showed that HOXB5 represses the apoptotic driver *BCL2L11* and promotes survival in the presence of etoposide, and that *miR10a* inhibits BCL6 and may thus play an oncogenic role in later stages of lymphomagenesis. Collectively, we characterize triple-hit B-cell line SC-1 and identify the aberrant expression of *HOXB5* and *miR10a*, both novel oncogenes in B-cell lymphoma.

## 1. Introduction

B-cell lymphomas form the majority of lymphomas and mainly derive from germinal-center (GC) or post-GC B cells [1]. Classification of lymphoid malignancies including B-cell lymphomas has been updated last year by the 5th edition of the World Health Organization [2]. Accordingly, the main types of B-cell lymphoma are Burkitt lymphoma (BL), diffuse large B-cell lymphoma (DLBCL), follicular lymphoma (FL), Hodgkin lymphoma (HL), mantle cell lymphoma (MCL), and plasma cell or multiple myeloma (MM). Aberrant rearrangements of the B-cell receptor genes may result in juxtapositional activation of proto-oncogenes—a hallmark of B-cell lymphoma [1]. Major oncogenes frequently associated with particular types of B-cell lymphoma are *BCL2* (FL, DLBCL), *BCL6* (DLBCL), *CCND1* (MCL, MM), and *MYC* (BL, DLBCL).

The oncogenes *BCL2*, *BCL6* and *MYC* are of special interest and drive deregulation of basic cellular processes including apoptosis, differentiation and proliferation [3,4,5]. Rearrangements of *BCL2* and *MYC* mostly juxtapose immunoglobulin genes (IG), while those of *BCL6* usually generate fusion genes with various partners [1,4]. *BCL2*, *MYC*, and/or *BCL6* are simultaneously rearranged in so-called double-hit and triple-hit lymphomas, respectively [6]. These tumor types have been grouped as high-grade B-cell lymphoma and are associated with poor prognosis, requiring special treatments [2,7,8]. Additional gene mutations and copy number alterations are frequently observed, and their investigation may assist in understanding lymphomagenesis and improving therapy [6,9,10].

Cell lines provide both experimental tools and renewable resources for mining novel oncogenes and targets to drive the development of improved therapeutic strategies [11,12,13,14]. Although double-hit and triple-hit B-cell lymphoma cell lines have been recently catalogued and reviewed [15], most such cell lines remain incompletely characterized genetically and bear unresolved complex alterations. Here, we describe cytogenetic analysis of a widely used B-cell lymphoma cell line SC-1 and report genes targeted by four major translocations. Accordingly, this cell line may now serve as a well-characterized model for triple-hit B-cell lymphoma and most notably for the novel oncogenes located at chromosomal position 17q21 whose investigation is detailed in this report.

## 2. Materials and Methods

### 2.1. Cell Lines and Treatments

B-cell lymphoma cell line SC-1 and control cell lines are held by the DSMZ (Braunschweig, Germany) and cultivated as described online (www.dsmz.de (accessed on 17 April 2023)). Authentication and absence of mycoplasma infection were confirmed as described previously [16,17]. Gene specific siRNA oligonucleotides, AllStars negative Control siRNA (siCTR), and miR10a RNA oligonucleotide were obtained from Qiagen (Hilden, Germany), and 100 pmol was transfected into 1 × 10^6^ cells by electroporation using the EPI-2500 impulse generator (Fischer, Heidelberg, Germany) at 350 V for 10 ms. Transfected cells were harvested after 20 h cultivation. For functional examinations, electroporated and etoposide-treated cells were analyzed by the IncuCyte S3 Live-Cell Analysis System (Essen Bioscience, Hertfordshire, UK). Etoposide was obtained from Sigma (Taufkirchen, Germany). For detection of apoptotic cells, we additionally used the IncuCyte Caspase-3/7 Green Apoptosis Assay diluted at 1:2000 (Essen Bioscience, Hertfordshire, UK).

### 2.2. Cytogenetic and Genomic Analyses

Karyotyping and fluorescence in situ hybridization (FISH) were performed as described previously [18]. Whole chromosome painting probes were obtained from Applied Spectral Imaging (Neckarhausen, Germany). RP11 BAC clones and fosmids were purchased from BacPac Resources, Children’s Hospital Oakland Research Institute (Emeryville, CA, USA) to analyze *BCL2* (RP11-215a20, 2270p21, 147g22), *MYC* (828I6, RP11-440n18, 125-A-17, RP11-288b17), *BCL6* (RP11-208n14, 211g3, 632m13, 67e18), *HOXB* (RP11-94I12, RP11-361k08, 6513b12, 0030b7, 2086b8, 5796a3, 388e10, 463m16), *IGH* (see Supplementary Figure S1), and *MIR17HG* (24M22, 383J16, 328H1). Probe DNA was harvested using the Big BAC DNA Kit (Princeton Separations, Adelphia, NJ, USA) and directly labelled by nick translation with dUTP-fluors (Dyomics, Jena, Germany). Fluorescent images were captured and analyzed with an Axio-Imager microscope (Zeiss, Göttingen, Germany) configured to a dual Spectral Imaging FISH system (Applied Spectral Imaging, Carlsbad, CA, USA).

For genomic profiling, DNA was prepared using the Qiagen Gentra Puregene Kit (Qiagen, Hilden, Germany). Labelling, hybridization, and scanning of HD Cytoscan arrays were performed at the Genome Analytics Facility, Helmholtz Centre for Infection Research (Braunschweig, Germany), according to the manufacturer’s protocols (Affymetrix, Waltham, MA, USA). Data were interpreted using the Chromosome Analysis Suite software version 3.1.0.15 (Affymetrix, Waltham, MA, USA).

### 2.3. Polymerase Chain Reaction (PCR) Analyses

Total RNA was extracted from cell line samples using TRIzol reagent (Invitrogen, Darmstadt, Germany). Primary human total RNA from selected cells/tissues was commercially obtained. We used RNA from peripheral blood mononuclear cells (PBC) and bone marrow (BM) obtained from Biochain/BioCat (Heidelberg, Germany) and RNA from CD34-positive hematopoietic stem cells (HSC), peripheral CD19-positive B cells, and CD3-positive T cells obtained from Miltenyi Biotec (Bergisch Gladbach, Germany). cDNA was synthesized from 1 µg RNA by random priming using Superscript II (Invitrogen, Darmstadt, Germany).

For detection of the common gene aberration *IGH::BCL2* we performed reverse transcription (RT)-PCR, using oligonucleotides IGH-JH and BCL2-mcr1 as reported previously [19]. To analyze *ETV6*, *BCL2*, *MYC*, *MBNL1*, and *BCL6*, we used the following oligonucleotides: ETV6-for 5′-AGGCCAATTGACAG-CAACAC-3′, ETV6-rev 5′-TGCACATTATCCACGGATGG-3′, BCL2-for 5′-GTGAACTGGGGGAGGATTGT-3′, BCL2-rev 5′-GGAGAAATCAAACAGAGGCC-3′, MYC-for 5′-TTGTACCTGCAGGATCTGAG-3′, MYC-rev 5′-AAGGTGATCCAGACTCTGAC-3′, MBNL1-for 5′-TTCAGCAGAAGAACATGGCC-3′, MBNL1-rev 5′-TGCAATTGCCACGTTGGTAC-3′, BCL6-for 5′-CTTAATCGTCTCCGGAGTCG-3′, BCL6-rev 5′-AGGATGCAGAATCCCTCAGG-3′. Fusion gene *MBNL1::BCL6* was detected combining MBNL1-for and BCL6-rev. The obtained PCR product was 138 bp long. All oligonucleotides were obtained from Eurofins MWG (Ebersberg, Germany). PCR products were generated using taqpol (Qiagen) and thermocycler TGradient (Biometra, Göttingen, Germany) analyzed by gel electrophoresis and documented with the Azure c200 Gel Imaging System (Azure Biosystems, Dublin, CA, USA).

HOX gene expression analysis was performed by a reported RT-PCR approach using degenerate oligonucleotides designed for amplification of diverse homeobox gene transcripts [20,21]. The generated PCR products were cloned into the vector pGEM-T Easy (Promega, Madison, WI, USA) and sequenced at Eurofins MWG.

Real-time quantitative (RQ)-PCR analysis was performed with the 7500 Real-time System, using commercial buffer and primer sets (Thermo Fisher Scientific, Darmstadt, Germany). For normalization of expression levels, we analyzed the transcript of TATA box binding protein (*TBP*). We used the ddCT method, and the obtained values are indicated as fold expression in relation to one sample, which was set to unity. Quantitative analyses were performed in biological and technical triplicates. Standard deviations are presented in the figures as error bars. Statistical significance was assessed by Student’s *t*-test, and the calculated *p*-values were indicated by asterisks (* *p* < 0.05, ** *p* < 0.01, *** *p* < 0.001, n.s.—not significant).

### 2.4. Protein Analysis

Western blots were generated by the semi-dry method. Protein lysates from cell lines were prepared using SIGMAFast protease inhibitor cocktail (Sigma, Taufkirchen, Germany). Proteins were transferred onto nitrocellulose membranes (Bio-Rad, München, Germany) and blocked with 5% dry milk powder dissolved in PBS. The following antibodies were used: alpha-Tubulin (Sigma, Taufkirchen, Germany), HOXB5 (Santa Cruz Biotechnology, Heidelberg, Germany), and BCL6 (Cell Signaling Technology, Danvers, MA, USA). For loading control, blots were reversibly stained with Poinceau (Sigma, Taufkirchen, Germany) and detection of alpha-Tubulin (TUBA) performed thereafter. Secondary antibodies were linked to peroxidase for detection by Western Lightning ECL (Perkin Elmer, Waltham, MA, USA). Documentation was performed using the digital system ChemoStar Imager (INTAS, Göttingen, Germany).

### 2.5. Expression Profiling and RNA-Seq Data Analyses

Expression profiling datasets of selected cell lines were generated by Dr. Robert Geffers (Genome Analytics, Helmholtz Centre for Infection Research, Braunschweig, Germany) and Dr. Andreas Rosenwald (University of Würzburg, Würzburg, Germany) using HG U133 Plus 2.0 gene chips (Affymetrix, Waltham, MA, USA). The primary data are available at Gene Expression Omnibus (www.NCBI.NLM.gov/GEO (accessed on 17 April 2023)) via GSE115191 and at BioStudies (www.ebi.ac.uk/biostudies/studies (accessed on 17 April 2023)) via S-BSST1073. After RMA-background correction and quantile normalization of the spot intensities, the profiling data were expressed as ratios of the sample mean and subsequently log2-transformed. Data processing was performed via R/Bioconductor using limma and affy packages. A heatmap for gene expression profiling data was generated using the public CLUSTER and TREEVIEW software (Michael Eisen, Berkeley, CA, USA). Public expression profiling datasets for cell lines (GSE57083), normal human myelopoiesis (GSE42519), and leukemia/lymphoma patients (GSE168422, GSE56311, GSE53786, GSE16455, GSE12453, GSE13576, GSE26713, GSE19554), in addition to RNA-seq dataset GSE69239 covering hematopoietic stem cells and lymphoid progenitor cells were all obtained from Gene Expression Omnibus. Gene expression profiling data were visualized using the associated online tool GEOR.

## 3. Results

### 3.1. Cytogenetic and Molecular Analysis of B-Cell Lymphoma Cell Line SC-1

**Figure 1:**
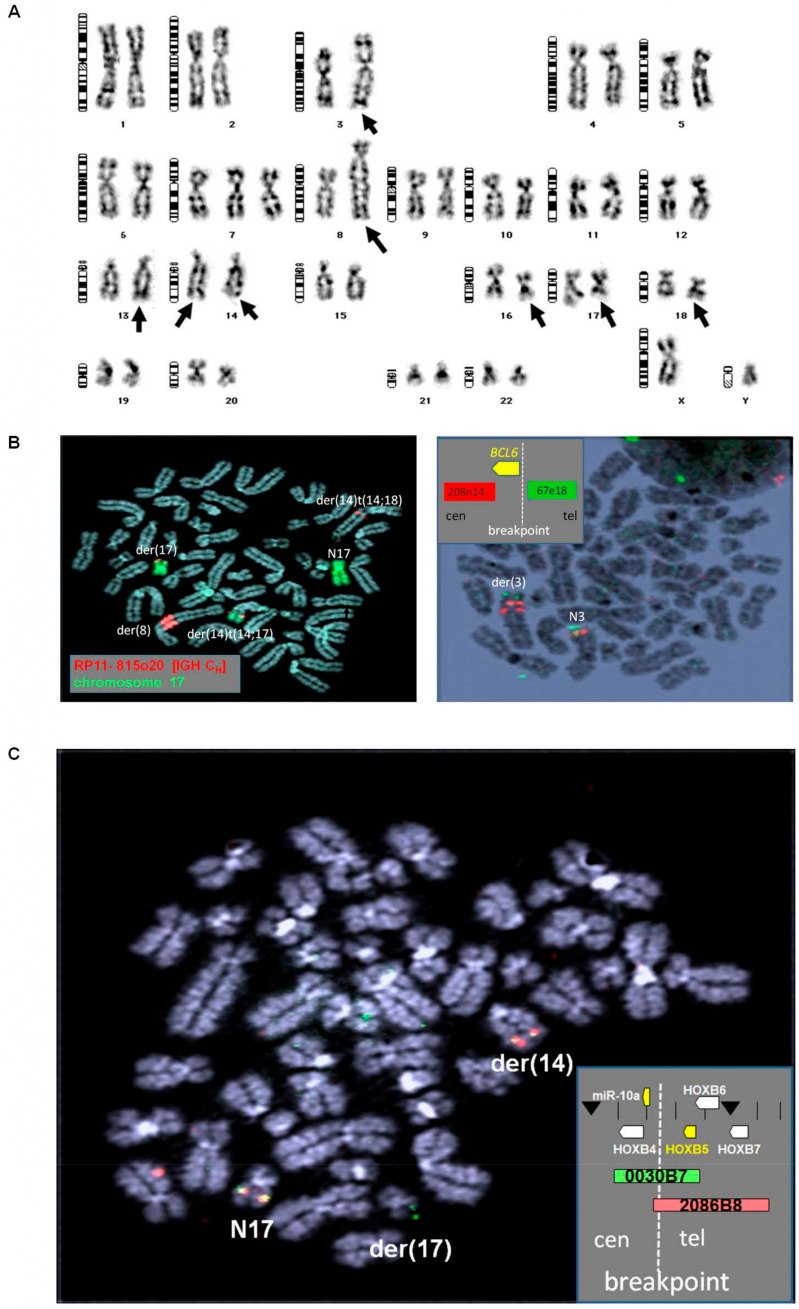
Cytogenetic characterization of SC-1. (**A**) Karyogram of SC-1. Altered chromosomes are indicated by arrows. (**B**) FISH analysis of *IGH* and chromosome 17 (**left**) and of *BCL6* (**right**) in SC-1. (**C**) FISH analysis of the *HOXB* gene cluster in SC-1. Used probes and their colors are indicated.

SC-1 is a B-cell lymphoma cell line derived from a 67-year-old male patient presenting with FL. Initial cytogenetic analysis revealed chromosomal rearrangement t(14;17)(q32;q21), representing an uncharacterized abnormality in hematopoietic malignancies [22,23]. In this study, we performed karyotyping (Figure 1A), FISH analysis (Figure 1B,C), and genomic profiling of SC-1 (Supplementary Figure S2) to reveal cytogenetic abnormalities in this interesting cell line which displayed a hyperdiploid karyotype bearing multiple rearrangements previously associated with B-cell lymphoma together with novel changes selected for more detailed analysis. The consensus karyotype is shown in Figure 1A and was as follows: 47,XY,der(3)dup(3)(q2?q2?)t(3;3)(q25;q27),+7,der(8)(pter->q12)qdp(8q24.1->8q24.2::14q32.3?->14q.3?::18q21->18q22)dup(8)(q24;q12)der(8)(q24.2->qter),hsr(13)(q3?1),der(14)t(14;17)(q32;q21),der(14)t(14;18)(q32;q21),del(16)(q13q31),der(17)hsr(17)(p11.2)t(14;17)(q32;q21),der(18)t(14;18)(q32;q21)/46 sl, –Y.

For more detailed analysis, we performed FISH and revealed the following four key oncogenic rearrangements depicted in Figure 1B,C: (1) t(14;18)(q32;q21) juxtaposing *IGH* and *BCL2* on der(14b) and der(18); (2) t(8;14)(q24;q32) juxtaposing *IGH* and *MYC* within an homogeneously staining region (hsr) on der(8) together with material from chromosome 18 including *BCL2*; (3) der(3)t(3;3)(q25;q27) involving partial duplication of the terminal long-arm region of chromosome 3 containing *MBNL1::BCL6* fusion—the first reported instance of this recurrent *BCL6* translocation in a cell line; and (4) t(14;17)(q32;q21)—a novel rearrangement involving der(14a) and der(17) juxtaposing *IGH* with 17q21, which hosts the *HOXB* gene cluster and is reported here for the first time.

Taken together, SC-1 represents a triple-hit B-cell lymphoma cell line, in which IGH rearrangements target FL-specific oncogene *BCL2*, *MYC*, as well as a novel target at 17q21, together with fusion of *BCL6* and *MBNL1*. In addition, cryptic amplification of the micro RNA gene cluster miR17-92 (*MIR17HG*) on chromosome 13q31 was detected on hsr(13) (Supplementary Figure S3).

### 3.2. Characterization of the Translocation Targets BCL2, MYC and BCL6

**Figure 2:**
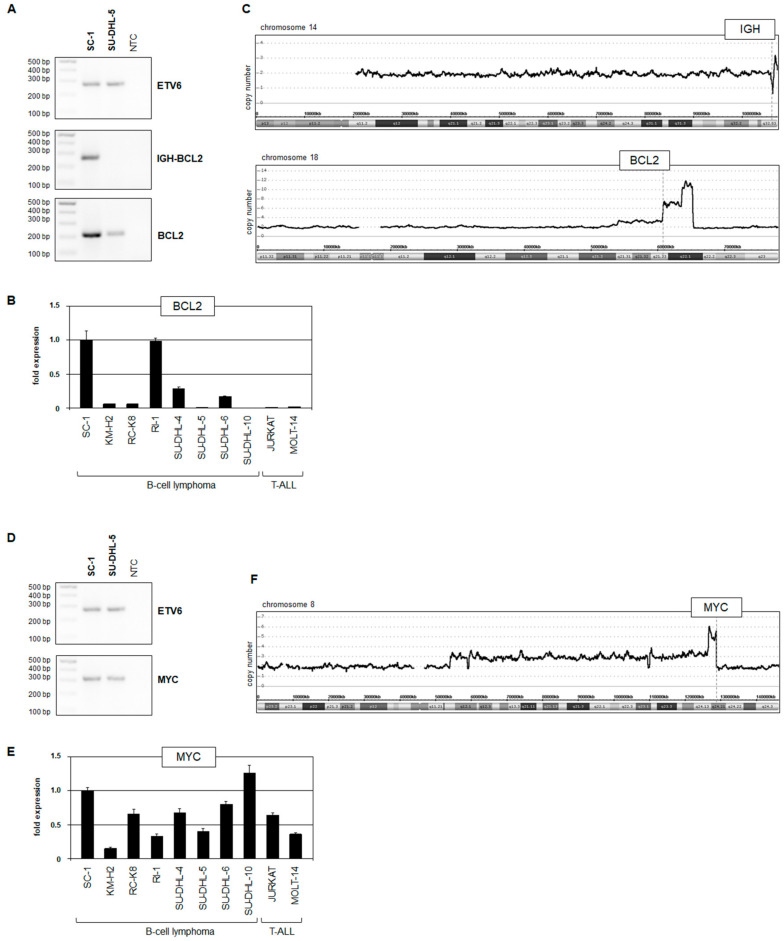
Analysis of IGH::BCL2 and IGH::MYC rearrangements. (**A**) RT-PCR analysis of *IGH::BCL2* and *BCL2*. *ETV6* served as positive control. (**B**) RQ-PCR analysis of *BCL2* in SC-1 and control cell lines. (**C**) Copy number analysis for SC-1 of chromosomes 14 and 18. The position of the genes *IGH* and *BCL2* are indicated. (**D**) RT-PCR analysis of *MYC*. *ETV6* served as positive control. (**E**) RQ-PCR analysis of *MYC* in SC-1 and control cell lines. (**F**) Copy number analysis for SC-1 of chromosome 8. The position of the gene *MYC* is indicated.

Using standardized BIOMED-2 primers, we confirmed the presence of *IGH::BCL2* juxtaposition in SC-1 by PCR, locating the *BCL2* breakpoint in the untranscribed downstream region (Figure 1A). Moreover, RT-PCR and RQ-PCR analyses showed strongly elevated expression levels of *BCL2* transcripts (Figure 2A,B), highlighting the demand for anti-apoptotic activity in this cell line. Copy number data for SC-1 demonstrated a combined small deletion and gain at the *IGH* locus at 14q32, which may result from both physiological and oncogenic genomic rearrangements (Figure 2C). The *BCL2* locus at 18q21 showed amplification (Figure 2C), which may contribute to enhanced *BCL2* expression.

RT-PCR and RQ-PCR analyses confirmed *MYC* expression in SC-1, which was, however, just slightly increased (Figure 2D,E). Nevertheless, copy number data for SC-1 also showed amplification of the *MYC* gene (Figure 2F), corresponding to the situation at BCL2. These similarities may underlie subsequent events of a three-way translocation.

**Figure 3:**
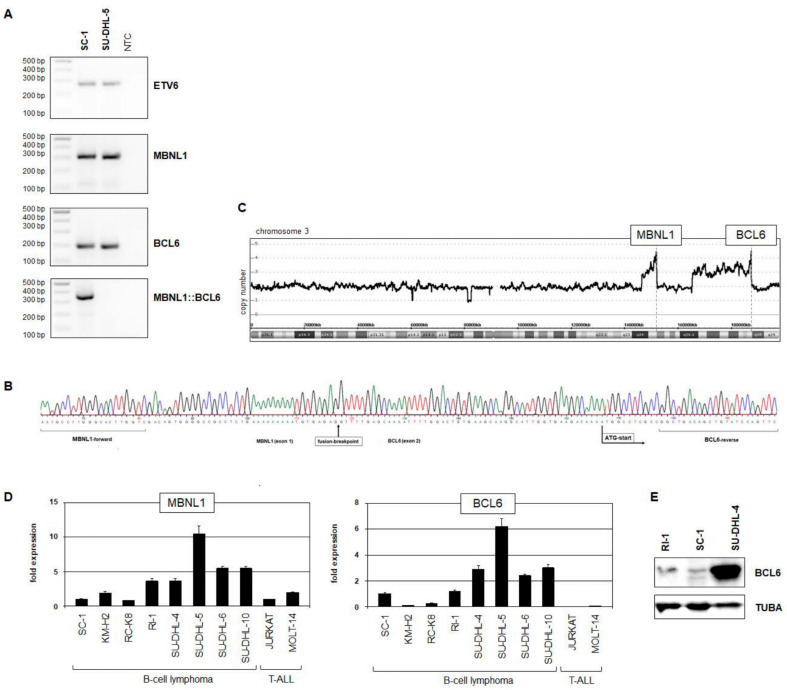
Analysis of MBNL1::BCL6 rearrangement. (**A**) RT-PCR analysis of *MBNL1*, *BCL6*, and *MBNL1::BCL6*. *ETV6* served as positive control. (**B**) Sequencing result of fusion gene *MBNL1::BCL6* in SC-1. Fusion breakpoint and ATG start are indicated. (**C**) Copy number analysis for SC-1 of chromosome 3. The position of the genes *MBNL1* and *BCL6* are indicated. (**D**) RQ-PCR analysis of *MBNL1* and *BCL6* in SC-1 and control cell lines. (**E**) Western blot analysis of BCL6. TUBA served as loading control.

Cytogenetic analysis indicated the presence of fusion gene *MBNL1::BCL6* generated via t(3;3)(q25;q27) in SC-1 (Figure 1). RT-PCR analysis demonstrated transcription of that fusion gene (Figure 3A), and sequence analysis of the generated RT-PCR product indicated location of the breakpoints in intron 1 of *MBNL1* and intron 1 of *BCL6*, fusing exon 1 and exon 2, respectively (Figure 3B). Copy number data for SC-1 showed amplification of both *MBNL1* and *BCL6* loci (Figure 3C), confirming the cytogenetic findings and suggesting that their copy number gain took place after the fusion event. Unexpectedly, the RQ-PCR analysis of *MBNL1* and *BCL6* discounted elevated expression levels (Figure 3D). Accordingly, Western blot analysis of BCL6 showed low protein levels in SC-1 and RI-1, while SU-DHL-4 cells exhibited high expression (Figure 3E). Thus, although SC-1 contains an amplified *BCL6*-fusion gene, this cell line failed to express elevated BCL6 levels predicted cytogenetically.

Taken together, SC-1 carries three major rearrangements resulting in the gene fusions *IGH::BCL2*, *IGH::MYC*, and *MBNL1::BCL6* which were characterized cytogenetically, genomically, and at the transcript level, thus, representing an exceptionally well characterized triple-hit B-cell lymphoma cell line.

### 3.3. Chromosomal Aberration t(14;17)(q32;q21) Targets the HOXB Gene Cluster

**Figure 4:**
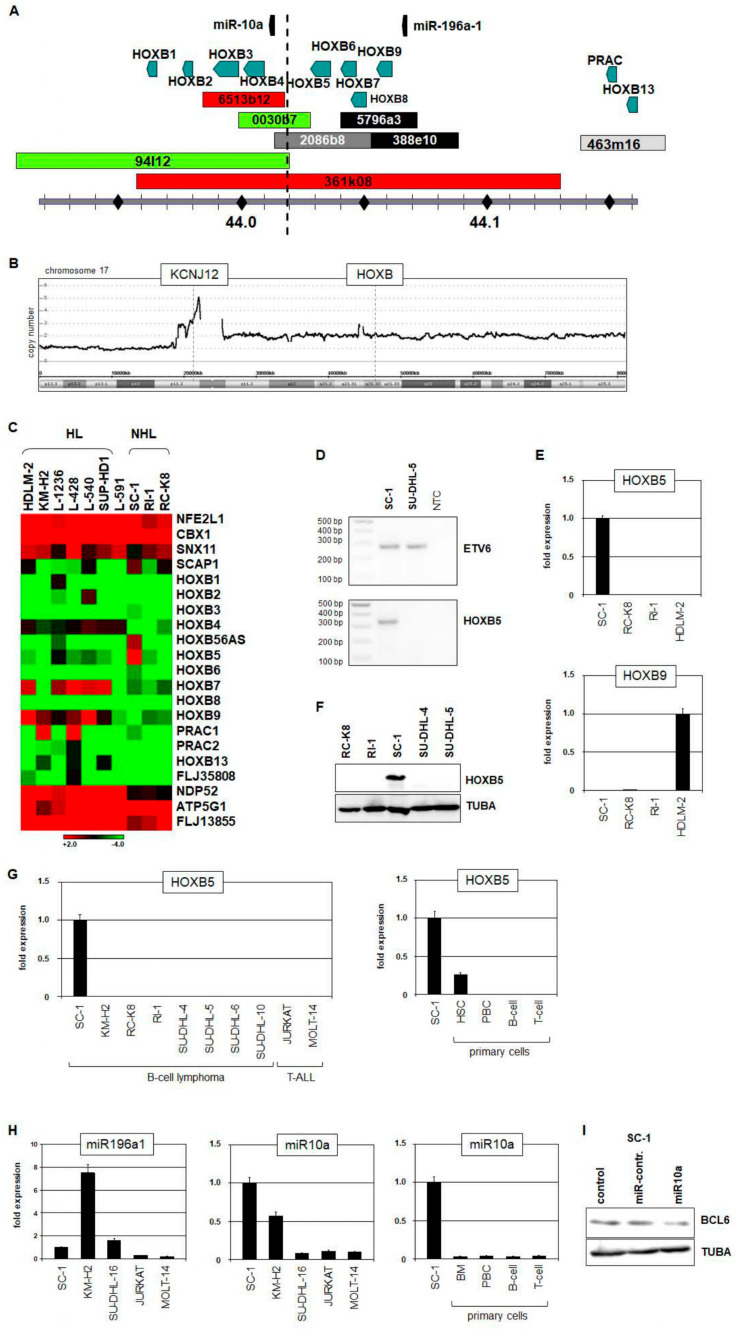
Analysis of IGH::HOXB rearrangement. (**A**) Diagram depicting the mapping strategy to detect the translocation breakpoint at 17q21. The location of genes, BACs, and fosmids are indicated. (**B**) Copy number analysis for SC-1 of chromosome 17. The position of the *HOXB* locus and of *KCNJ12* are indicated. (**C**) Heatmap showing gene expression profiling data of *HOXB* cluster members and flanking genes in SC-1 and control cell lines. (**D**) RT-PCR analysis of *HOXB5*. *ETV6* served as positive control. (**E**) RQ-PCR analysis of *HOXB5* (above) and *HOXB9* (below) in SC-1 and control cell lines. (**F**) Western blot analysis of HOXB5 in SC-1 and control cell lines. TUBA served as loading control. (**G**) RQ-PCR analysis of *HOXB5* in cell lines (left) and primary cells (right). (**H**) RQ-PCR analysis of *miR196a1* and *miR10a* in cell lines and primary cells. (**I**) Western blot analysis of BCL6 in SC-1 cells electroporated with additional miR10a (right). TUBA served as loading control.

In addition to the triple-hit rearrangements described above, SC-1 bears a novel chromosomal aberration t(14;17)(q32;q21) whose targets are yet to be identified and characterized [22]. To address this question, we performed chromosomal mapping by repeated rounds of FISH analysis and located the breakpoints of *IGH* at 14q32 (Supplementary Figure S1) and of the *HOXB* gene cluster at 17q21 (Figure 1C and Figure 4A). Our genomic data excluded copy number alterations at chromosomal position 17q21 but indicated amplification of *KCNJ12* at 17p12, which was overexpressed in SC-1 (Figure 4B, Supplementary Table S1). To identify potentially deregulated target genes located near the *HOXB* breakpoint, we adopted a previously reported RT-PCR strategy using degenerate oligonucleotides to amplify homeobox gene transcripts (Supplementary Figure S4) and gene expression profiling of SC-1 and nine control cell lines (Figure 4C). Both assays highlighted aberrant activation of *HOXB5*. Furthermore, public gene expression profiling data for 114 hematopoietic cell lines (Supplementary Figure S5), in addition to RT-PCR, RQ-PCR, and Western blot analyses, confirmed *HOXB5* expression exclusive to SC-1 (Figure 4D–G). Together, these data show that *HOXB5* is an activated target gene of t(14;17)(q32;q21). Recently, we reported aberrant *HOXB9* expression in HL which was confirmed by gene expression profiling analysis (Figure 4C) and tested negative in SC-1 by RQ-PCR (Figure 4E) [21]. Here, we identified *HOXB5* representing another member of the *HOXB* gene cluster aberrantly expressed in B-cell lymphoma.

To study *HOXB5* expression in B-cell lymphoma patients, we exploited public gene expression profiling datasets (Supplementary Figure S6). The data revealed *HOXB5* overexpression in subsets of BL, FL, MCL, and MM while discounting significant overexpression in HL, DLBCL, precursor B-cell acute lymphoid leukemia (ALL), and T-ALL. Subsets of BL, FL, and MM patients showed the most prominent activation (Supplementary Figure S6), indicating that *HOXB5* may play an important role in these B-cell malignancies.

In addition to 10 homeobox genes, the *HOXB* gene cluster locus contains two micro-RNA genes, namely *miR10a* and *miR196a1*, which were not included in the screenings described above (Figure 4A). RQ-PCR analysis of their primary transcripts in selected cell lines revealed elevated expression of *miR10a* in SC-1, while *miR196a1* was not conspicuously activated in this cell line, in direct contrast to HL cell line KM-H2 (Figure 4H). Furthermore, the expression level of *miR10a* was enhanced in SC-1 when compared to selected primary hematopoietic cells (Figure 4H). These data suggest that *miR10a* is aberrantly activated by chromosomal translocation t(14;17)(q32;q21) in SC-1 such as the immediately neighboring *HOXB5*. Recently, Fan and colleagues reported that *miR10a* targets BCL6 for suppression in DLBCL [24]. Therefore, we electroporated additional *miR10a* oligonucleotides into SC-1 cells and performed Western blot analysis. The results showed reduced BCL6 protein after this treatment (Figure 4I), confirming the suppressive function of *miR10a* in SC-1, as reported in DLBCL by these authors.

Collectively, our data show that chromosomal aberration t(14;17)(q32;q21) mediates activation of both homeobox gene *HOXB5* and micro-RNA gene *miR10a* in triple-hit B-cell lymphoma cell line SC-1, and that aberrant overexpression of *HOXB5* was also detected in subsets of BL, FL, and MM patients.

### 3.4. Functional Analysis of HOXB5 in B-Cell Lymphoma

**Figure 5:**
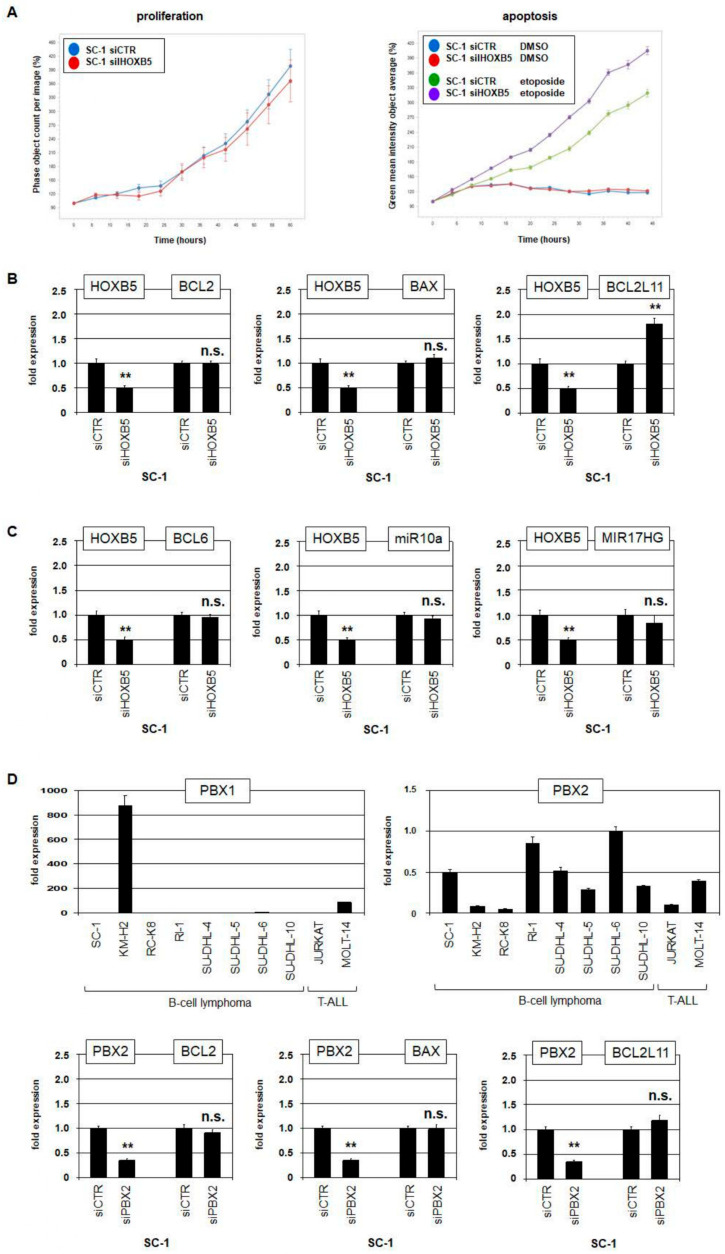
Functional analysis of HOXB5. (**A**) Live-cell imaging analysis of SC-1 cells treated for knockdown of *HOXB5* (left) and additionally with 100 µM etoposide dissolved in DMSO (right). Phase object count represents the cells corresponding to proliferation (left). Green intensity represents dying cells corresponding to the number of apoptotic cells (right). (**B**,**C**) RQ-PCR analysis of six target gene candidates in SC-1 cells treated for knockdown of *HOXB5*. (**D**) RQ-PCR analysis of SC-1 and control cell lines for *PBX1* (left) and *PBX2* (right). RQ-PCR analysis of SC-1 cells treated for knockdown of *PBX2* (below). Statistical significance was assessed by Student’s *t*-test, and the calculated *p*-values were indicated by asterisks (** *p* < 0.01, n.s.—not significant).

While aberrant activation of *miR10a* reduced BCL6 in B-cell lymphoma, the role of *HOXB5* remained unclear. Therefore, we performed live-cell imaging analysis of SC-1 cells treated for siRNA-mediated knockdown of *HOXB5*. We observed no impact on cell proliferation, while additional treatment with etoposide indicated a role for HOXB5 in supporting cell survival (Figure 5A). We concluded that HOXB5 may activate or suppress transcription of particular regulators of apoptosis and, accordingly, analyzed SC-1 cells treated for *HOXB5* knockdown by RQ-PCR. While *BCL2* and *BAX* remained unperturbed, we found that HOXB5 inhibited expression of the apoptotic driver *BCL2L11/BIM* (Figure 5B). In addition, we analyzed the potential impact of HOXB5 on expression of *BCL6*, *miR10a*, and *MIR17HG* (Figure 5C). However, these genes showed no significant alteration of their activity after *HOXB5* knockdown. Of note, *MIR17HG* was targeted in SC-1 by a focal genomic amplification at 13q21 (Supplementary Figure S3) and showed enhanced expression (Supplementary Table S1), indicating oncogenic activity of this micro-RNA gene in SC-1 cells and triple-hit B-cell lymphoma.

HOX proteins are able to interact with cofactors of the TALE-class of homeodomain proteins including PBX [25,26]. RQ-PCR and gene expression profiling analysis of selected cell lines excluded *PBX1* activity in SC-1 while *PBX2* was clearly expressed (Figure 5D, Supplementary Table S1). However, siRNA-mediated knockdown of *PBX2* in SC-1 showed no impact on expression levels of *BCL2*, *BAX*, and *BCL2L11* (Figure 5D), discounting a role for PBX2 as a cofactor of HOXB5 in BCL2L11 regulation.

### 3.5. HOXB5 and ZNF521 in Stem Cells and B-Cell Lymphoma

**Figure 6:**
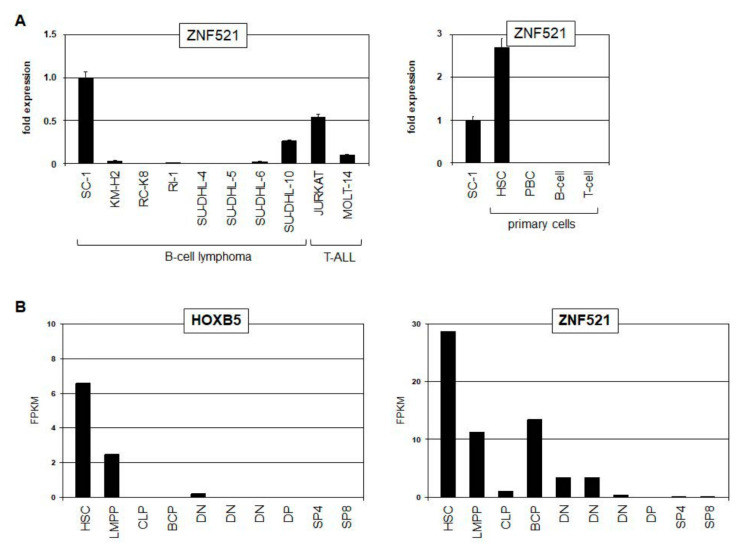

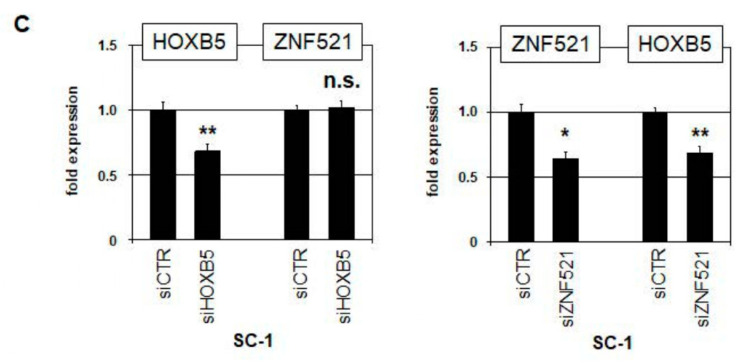
Regulatory relationships between HOXB5 and ZNF521. (**A**) RQ-PCR analysis of *ZNF521* in SC-1 and control cell lines (**left**) and primary cells (**right**). (**B**) Public RNA-seq gene expression data obtained from dataset GSE69239 for *HOXB5* (**left**) and *ZNF521* (**right**) in primary hematopoietic stem cells (HSCs), lymphoid and myeloid primed progenitors (LMPPs), common lymphoid progenitors (CLP), B-cell progenitors (BCPs), double negative thymocytes (DN), double positive thymocytes (DP), CD4 single positive thymocytes (SP4), and CD8 single positive thymocytes (SP8). (**C**) RQ-PCR analysis of SC-1 cells treated for knockdown of *HOXB5* (**left**) and *ZNF521* (**right**). Statistical significance was assessed by Student’s *t*-test, and the calculated *p*-values were indicated by asterisks (* *p* < 0.05, ** *p* < 0.01, n.s.—not significant).

*HOXB5* reportedly plays a basic role in early hematopoietic cell differentiation, while aberrant expression suppressed B-cell differentiation [27,28]. Comparative gene expression profiling analysis of SC-1 versus seven B-cell lymphoma control cell lines demonstrated elevated *HOXB5* activity and revealed high expression levels of *ZNF521* in SC-1 (Supplementary Table S1). The RQ-PCR analysis of *ZNF521* in cell lines confirmed enhanced expression levels in SC-1 (Figure 6A). Furthermore, SC-1 expressed significant levels of *ZNF521* as compared to HSCs (Figure 6A). This zinc-finger transcription factor plays a role in hematopoietic progenitors and is associated with pre-B-ALL [29,30]. Public RNA-seq data from hematopoietic stem cells and lymphoid progenitor cells and gene expression profiling data from myeloid progenitors showed elevated co-expression of *ZNF521* and *HOXB5* in early stages (Figure 6B and Figure S7), supporting their described role in stem and progenitor cell differentiation and indicating mutual regulation. SiRNA-mediated knockdown experiments demonstrated that ZNF521 activated *HOXB5* expression without reciprocal action (Figure 6C). Thus, both *HOXB5* and *ZNF521* are active in hematopoietic stem and progenitor cells and exhibit regulatory connections. These data support the conclusion that aberrant *HOXB5* expression may deregulate differentiation processes in B-cell lymphoma.

## 4. Discussion

**Figure 7:**
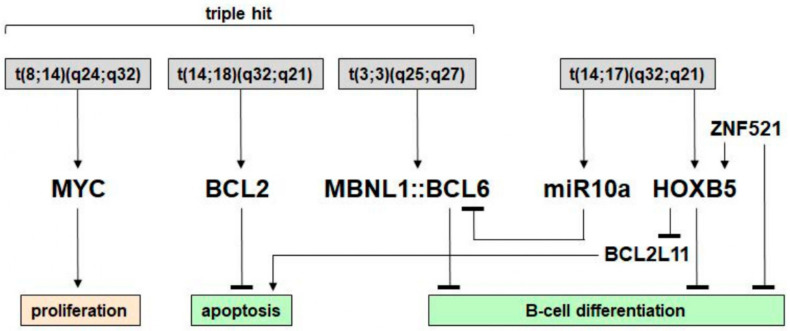
Summary of chromosomal aberrations and their gene targets in SC-1.

In this study, we describe the characterization of chromosomal aberrations and their target genes in a standard B-cell lymphoma cell line model SC-1 and summarize our results in Figure 7. Identification and examination of t(14;18)(q32;q21), t(8;14)(q24;q32), and t(3;3)(q25;q27) demonstrated the presence of fusion genes *IGH::BCL2*, *IGH::MYC*, and *MBNL1::BCL6*, respectively. Thus, SC-1 invites classification as a triple-hit B-cell lymphoma cell line. Double-hit and triple-hit B-cell lymphomas are generally associated with poor prognosis although a more favorable outcome has been reported for a patient with *MBNL1::BCL6* in whom absence of BCL6 overexpression was also noted [6,9,31]. As well as serving as tools for pathological investigation, well-characterized cell lines, such as SC-1, can serve as models to design and evaluate therapies targeted to the specific lymphoma subtypes revealed by their molecular analysis [12,13,14,15]. Most notably, we report a new *IGH* rearrangement, t(14;17)(q32;q21), mapping the 17q21 breakpoint inside the *HOXB* gene cluster near *HOXB5* and *miR10a* which were both aberrantly activated and, accordingly, deemed target genes. Analysis of patient data demonstrated aberrant expression of *HOXB5* in BL, FL, and MM, endorsing the clinical relevance of our findings. Supplementary Figure S8 summarizes these four major chromosomal aberrations, proposing an order of their origin in SC-1. At first, both alleles of *IGH* were rearranged targeting the loci of *MYC*, *HOXB5/miR10a*, and *BCL2*. *MYC*, *BCL2*, and *MIR17HG* were subsequently amplified. Finally, the genes *MBNL1* and *BCL6* were fused.

Our data further showed that HOXB5 enhanced survival by suppression of pro-apoptotic gene *BCL2L11*. This observation is supported by experiments performed in hepatoma cell lines [32]. Interestingly, BCL2L11 is reportedly repressed by *miR10a* in neurons associated with Parkinson’s disease and by *miR17* in B-cell development [33,34], highlighting a role for these genes in SC-1 and more generally in B-cell lymphoma. Of note, *miR10a* and *miR17* play physiological and oncogenic roles in normal and malignant hematopoiesis [35]. Here, we have shown that *miR10a* was coactivated by t(14;17)(q32;q21) together with *HOXB5*. Recently, we reported aberrant co-expression of *HOXB9* and *miR196a* in HL, which was confirmed in this study [21,36]. Intriguingly, our collective findings show that while neighboring homeobox gene *HOXB9* and microRNA gene *miR196a* may promote HL, the analogous pair described here—*HOXB5* and *miR10a*—playing comparable roles in non-HLs, raise the possibility that their close genomic proximities may reflect physiological cooperation in their respective developmental pathways.

PLZF/ZBTB16 interacts with HOXB5 in developing limb and together with BCL6 in immune cells [37,38]. Therefore, impacting the function of BCL6 via HOXB5 or *miR10a* as shown here, may well be of tumorigenic relevance, both in SC-1 cells and more generally in B-cell lymphoma [24]. BCL6 regulates the differentiation of B cells and plays important developmental roles in GC B cells. Later, in B-cell development, *BCL6* is repressed by PRDM1 and other factors [39]. Thus, HOXB5 and *miR10a* may deregulate B-cell differentiation via BCL6.

Zhang and colleagues have shown that HOXB5 represses B-cell master genes including *BCL11A*, *EBF1*, and *FOXP1*. Consequently, aberrant expression of *HOXB5* reprograms B cells into early T cells [28]. Furthermore, *HOXB5* plays a role in stem cell (de)regulation, while *ZNF521* also serves as an hematopoietic stem cell factor [27,29]. We have shown that ZNF521 activates *HOXB5* expression, supporting a potential stem cell role oncogenically reactivated by *HOXB5* in B-cell lymphoma. Interestingly, our data demonstrate that *HOXB5* and *ZNF521* are co-expressed in myeloid progenitors, while others have reported their aberrant expression in corresponding acute myeloid leukemia [40,41]. Together, our data may suggest that aberrantly activated *HOXB5* plays a prominent role in deregulation of B-cell development. Deregulated homeobox genes from the *HOXA* and *HOXB* clusters or members of the NKL- and TALE-classes operate as oncogenes in most types of hematopoietic malignancies. These developmental regulators impact differentiation processes, apoptosis and proliferation performing various roles in cancerogenesis [21,36,42,43]. Here, we added *HOXB5* to that list of homeo-oncogenes.

Finally, we detected two amplicons in SC-1, targeting overexpressed genes *MIR17HG* at 13q31 and *KCNJ12* at 17p12. *MIR17HG* encodes several micro-RNAs operating as well-known oncogenes in hematopoietic malignancies including B-cell lymphoma [44]. *KCNJ12* encodes a potassium channel protein which is reportedly deregulated and mutated in solid cancer and chronic myeloid leukemia, respectively [45,46]. However, its role in B-cell lymphoma remains to be investigated.

Taken together, we identified several deregulated genes in triple-hit B-cell lymphoma cell line SC-1, including *BCL2*, *BCL2L11*, *BCL6*, *HOXB5*, *KCNJ12*, *miR10a*, *miR17*, and *ZNF521*, together with new regulatory interconnections. It seems that these rearrangements occur sequentially and involve fine-tuning of previous oncogene upregulations during lymphomagenic evolution, potentially rendering these interconnections both pathogenically informative and therapeutically relevant. In short, our data highlight new cancer targets and their interplay and may light the way towards preclinical development of novel targeted therapies in intractable high-grade B-cell lymphoma, as modelled here by SC-1 cells.

## Data Availability

The information on the datasets generated or analyzed during this study are included in this published article and its Supplementary Information files. The accession codes of all publicly available data are given in the Material and Methods section.

## Supplementary Materials

The following supporting information can be downloaded at: https://www.mdpi.com/article/10.3390/biomedicines11061758/s1.
