# Supplementary material for: Chromosomal Aberration t(14;17)(q32;q21) Simultaneously Activates HOXB5 and miR10a in Triple-Hit B-Cell Lymphoma
Source: Biomedicines. 2023 Jun 19;11(6):1758. doi: 10.3390/biomedicines11061758 (PMC10296542; doi:10.3390/biomedicines11061758)
Supplement: Supplementary file 1 [file biomedicines-11-01758-s001.zip › biomedicines-2453159-supplementary.pdf]

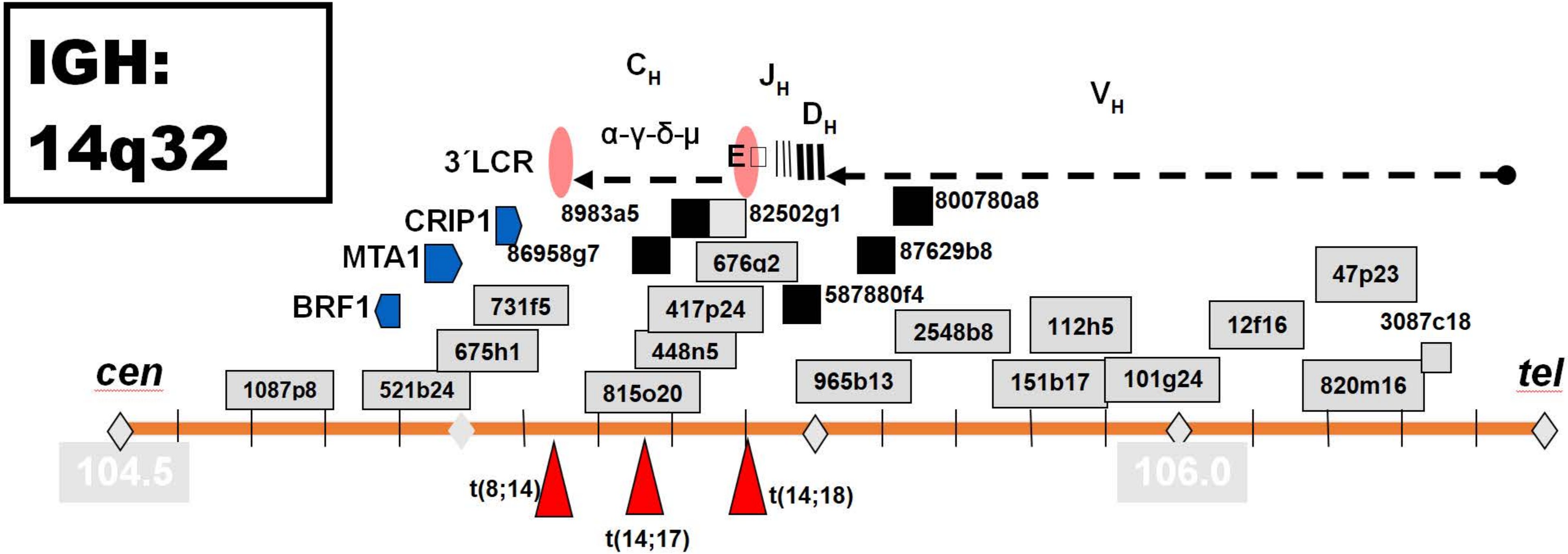

Supplementary Figure 1: FISH analysis of the IGH locus at 14q32.

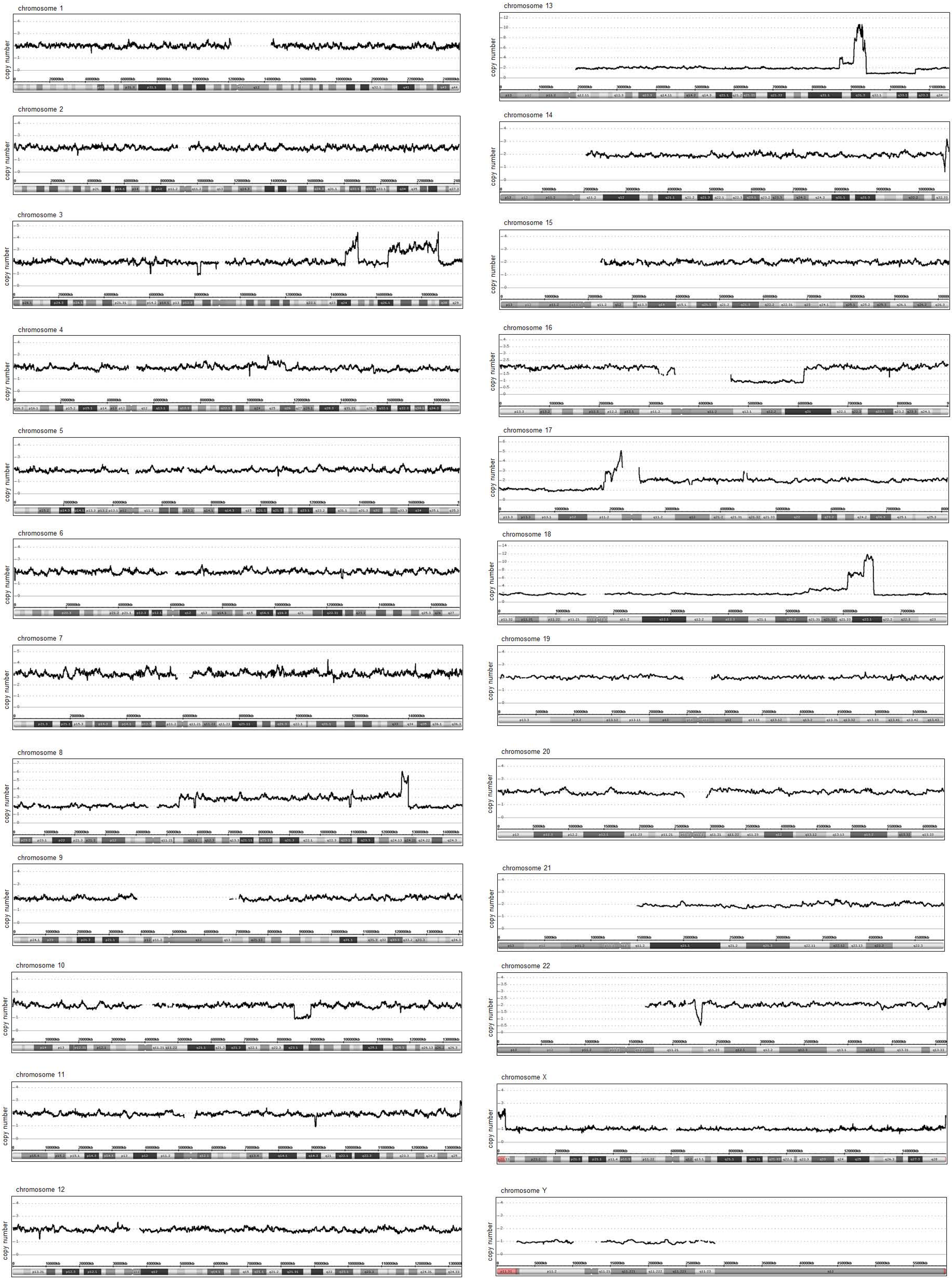

Supplementary Figure 2: Copy number analysis of SC-1.

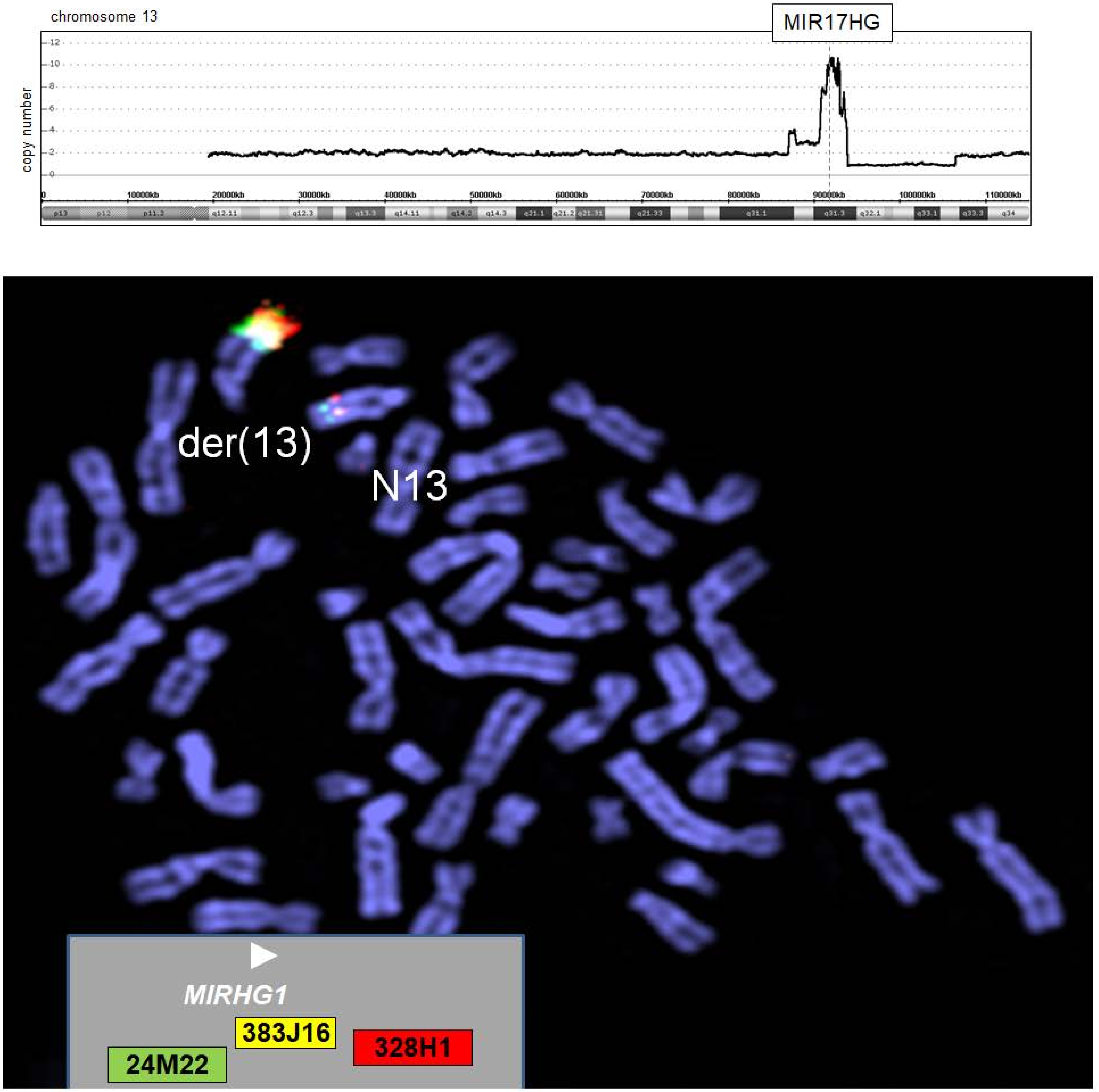

Supplementary Figure 3: Copy number data of SC-1 for MIR17HG (above). FISH analysis of MIR17HG in SC-1 (below). Used probes and their colors are indicated.

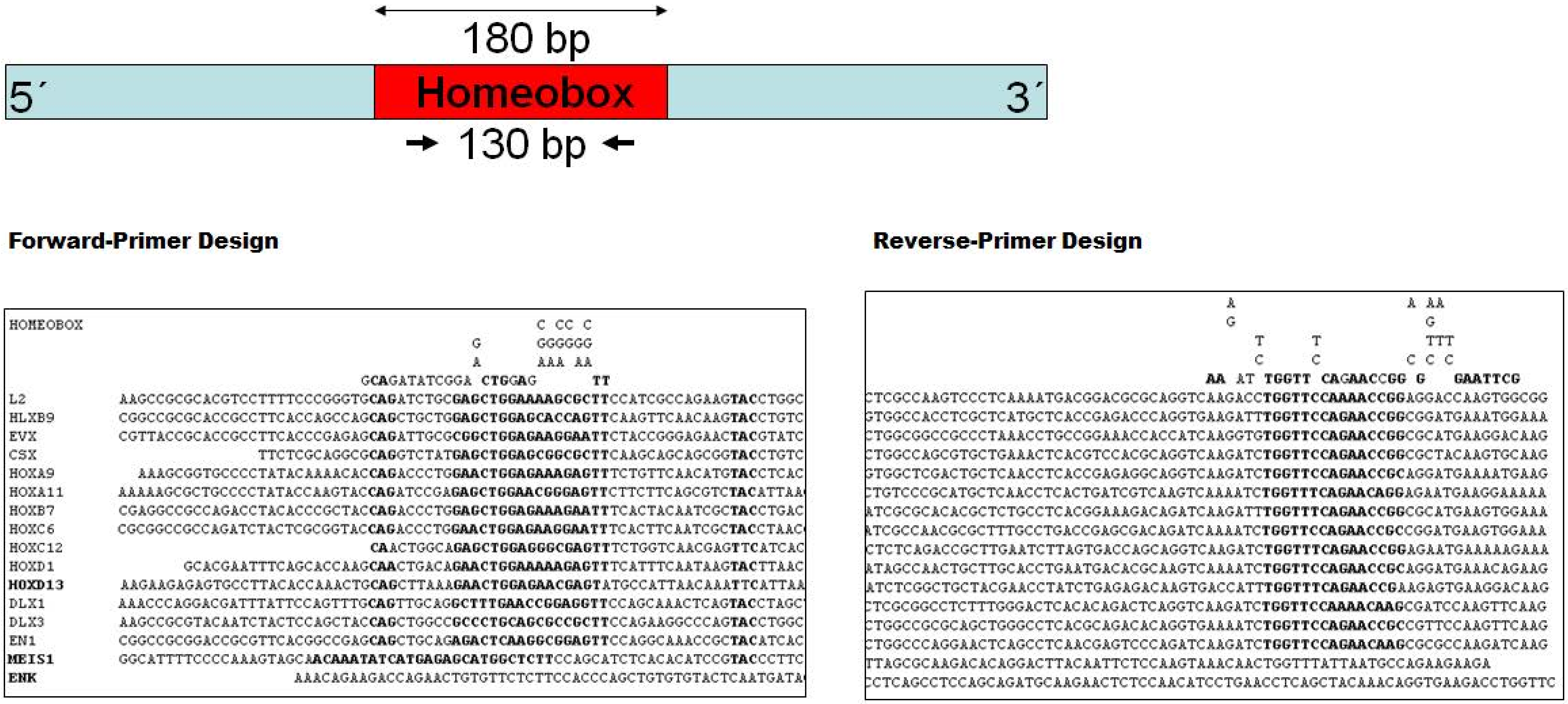

SC-1

Clone No	Homeobox gene	Frequencies		
1	HOXA4	HOXB5	5x	31 %
2	**HOXB6**	HOXA4	4x	
3	HLXB9	HLXB9	4x	
4	HOXA4	HOXA1	2x	
5	HOXA1	**HOXB6**	1x	6 %
6	HOXA4			
7	**HOXB5**			
8	HLXB9			
9	**HOXB5**			
10	HOXA4			
11	**HOXB5**			
12	HLXB9			
13	**HOXB5**			
14	HOXA1			
15	HLXB9			
16	**HOXB5**			

Supplementary Figure 4: RT-PCR screening approach and results for expressed homeobox genes in SC-1 using degenerate oligonucleotides.

Hematopoietic cell lines (GSE57083)

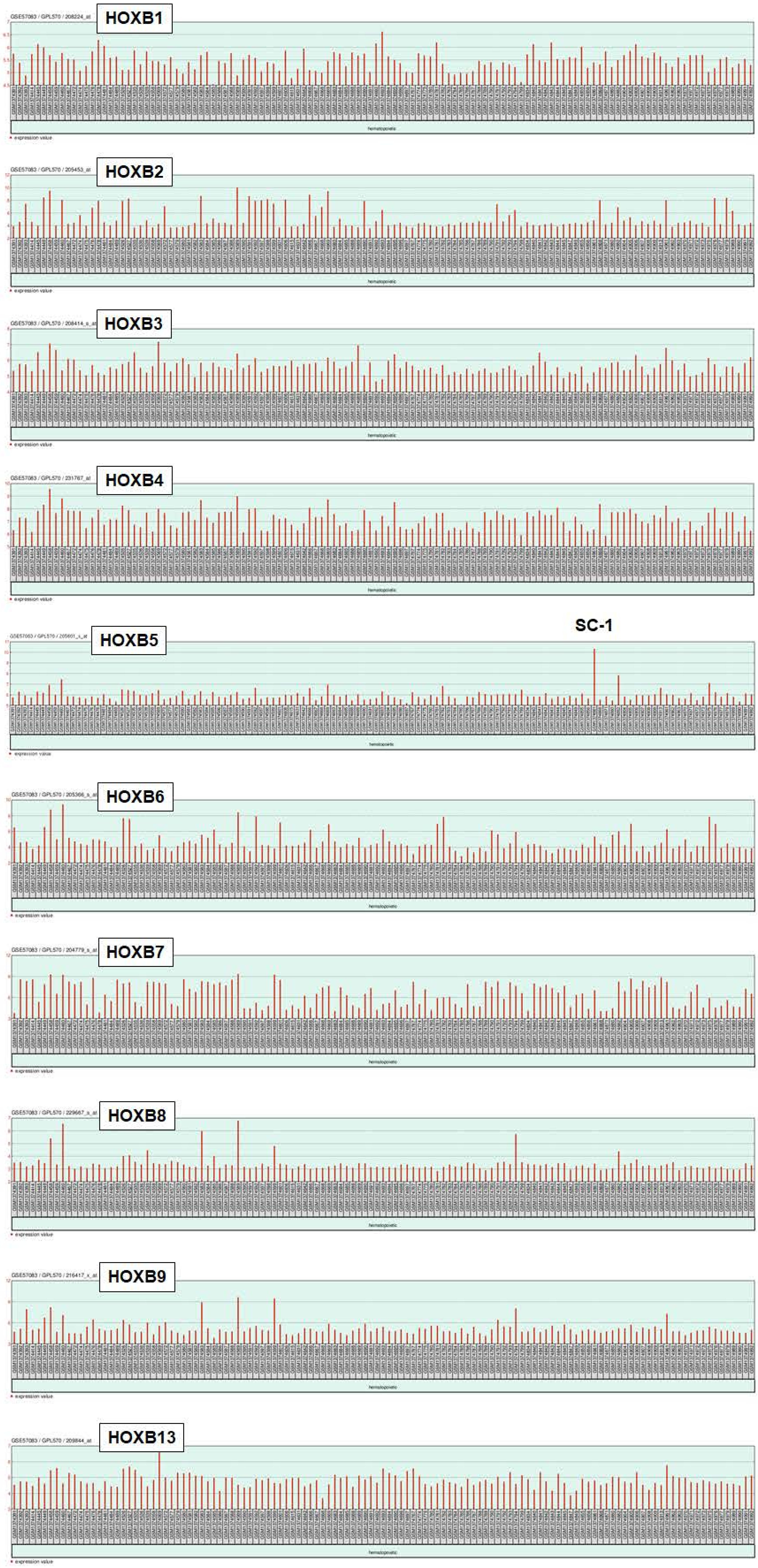

GSM1374391	Bx030_019 HG2
GSM1374392	AMO1 ghp280606
GSM1374393	ARH77 ghp280606
GSM1374414	Bx030_010 HG2
GSM1374445	CCRFCEM
GSM1374449	Bx030_001 HG2
GSM1374458	CTV-1 ghp221105
GSM1374459	Daudi ghp221105
GSM1374460	Bx030_020 HG2
GSM1374467	DOHH-2 ghp221105
GSM1374472	EHEB ghp221105
GSM1374474	EA10068_111347
GSM1374475	EA10068_111339
GSM1374476	Bx030_003 HG2
GSM1374478	Bx030_021 HG2
GSM1374481	FARAGE
GSM1374484	Bx030_016 HG2
GSM1374489	HBL1
GSM1374526	HEL 92.1.7 ghp221105
GSM1374527	EA10068_111346
GSM1374535	HL60
GSM1374536	HL 60 ghp221105
GSM1374539	HLY-1
GSM1374558	HT ghp280606
GSM1374569	HUT78
GSM1374572	IM9 ghp280606
GSM1374577	JEKO ghp280606
GSM1374579	JJN3 ghp280606
GSM1374580	Jurkat ghp280606
GSM1374581	JVM-2 ghp221105
GSM1374582	JVM-3 ghp221105
GSM1374583	K562 ghp231105
GSM1374584	Bx030_022 HG2
GSM1374585	Karpas1106 ghp280606
GSM1374586	Bx030_011 HG2
GSM1374587	KARPAS-231 ghp231105
GSM1374588	KARPAS-422 ghp231105
GSM1374589	Karpas620 ghp280606
GSM1374590	Bx030_014 HG2
GSM1374591	EA10068_111342
GSM1374592	Bx030_023 HG2
GSM1374597	KG-1 ghp231105
GSM1374598	KG-1a ghp231105
GSM1374599	Bx030_006 HG2
GSM1374601	KMS12BM ghp280606
GSM1374606	KU812 ghp231105
GSM1374615	L363 ghp280606
GSM1374631	LP1 ghp280606
GSM1374642	Bx030_017 HG2
GSM1374666	EA08079_89055_ME1
GSM1374667	MEC1 ghp280606
GSM1374668	Bx030_024 HG2
GSM1374669	EA10068_111348
GSM1374683	ML-2 ghp231105
GSM1374684	EA08079_89044_MM1.R
GSM1374685	EA08079_89045_MM1.S
GSM1374688	Bx030_015 HG2
GSM1374689	MOLM13
GSM1374690	Bx030_025 HG2
GSM1374691	MOLP8 ghp280606
GSM1374692	Bx030_026 HG2
GSM1374693	Bx030_009 HG2
GSM1374694	MV-4-11 ghp231105
GSM1374695	Namalwa ghp231105
GSM1374696	Bx030_005 HG2
GSM1374697	NB4
GSM1374767	NCLH929 ghp280606
GSM1374774	Bx030_007 HG2
GSM1374775	NU-DHL-1
GSM1374780	Bx030_002 HG2
GSM1374781	Bx030_027 HG2
GSM1374782	OCI-AML5 ghp280606
GSM1374783	EA08079_89046_OCI-LY1
GSM1374784	EA08079_89047_OCI-LY10
GSM1374785	EA08079_89048_OCI-LY18
GSM1374786	OCI-LY19 ghp280606
GSM1374787	EA08079_89049_OCI-LY3
GSM1374788	EA08079_89050_OCI-LY4
GSM1374789	EA08079_89051_OCI-LY7
GSM1374790	EA08079_89052_OCI-LY8
GSM1374791	Bx030_028 HG2
GSM1374792	EA10068_111343
GSM1374793	EA08079_89053_OCI-MY1
GSM1374794	EA08079_89054_OCI-MY5
GSM1374799	OPM-2 ghp280606
GSM1374834	EA10068_111340
GSM1374840	Bx030_029 HG2
GSM1374841	Raji ghp231105
GSM1374842	Raji ghp280606
GSM1374843	Bx030_012 HG2
GSM1374844	Ramos ghp231105
GSM1374845	RC-K8
GSM1374847	Bx030_004 HG2
GSM1374849	RI-1
GSM1374855	RPMI8226 ghp280606
GSM1374856	EA10068_111349
GSM1374861	SC1 ghp280606
GSM1374868	EA10068_111345
GSM1374871	Bx030_008 HG2
GSM1374880	SKM-1 ghp231105
GSM1374882	SKMM2 ghp150606
GSM1374904	SUDHL10
GSM1374905	SUDHL2
GSM1374906	SU-DHL-4 ghp231105
GSM1374907	Bx030_013 HG2
GSM1374908	Bx030_030 HG2
GSM1374909	SUDHL8
GSM1374913	Bx030_031 HG2
GSM1374961	TF-1 ghp231105
GSM1374962	THP-1 ghp231105
GSM1374963	TMD8
GSM1374970	U266B1 ghp150606
GSM1374971	U2932
GSM1374972	U-2940
GSM1374973	U-698-M
GSM1374975	U937 ghp280606
GSM1374976	EA10068_111344
GSM1374977	ULA
GSM1374979	EA10068_111341
GSM1374989	WILL-1
GSM1374990	WILL-2
GSM1374991	WSU-DCL2 ghp280606
GSM1374992	WSU-NHL ghp231105

Supplementary Figure 5: Expression profiling data for HOXB genes in 114 hematopoietic cell lines including SC-1 (dataset GSE57083).

BCP-ALL (GSE13576)

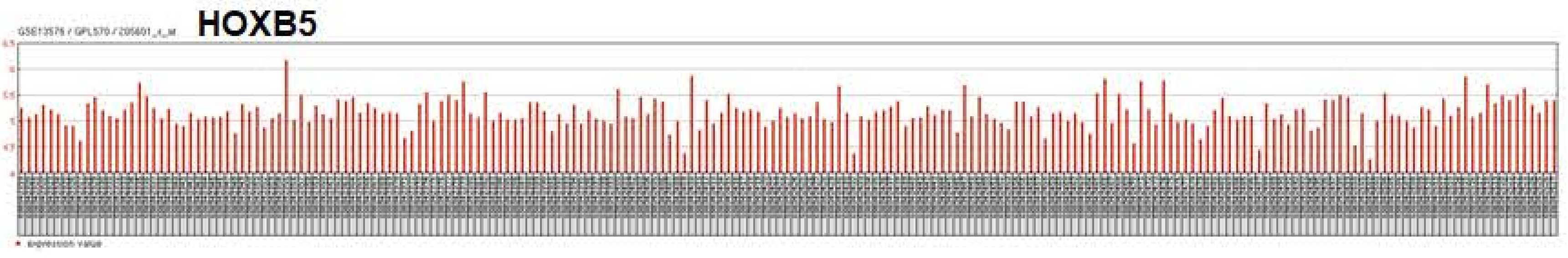

BL (GSE168422)

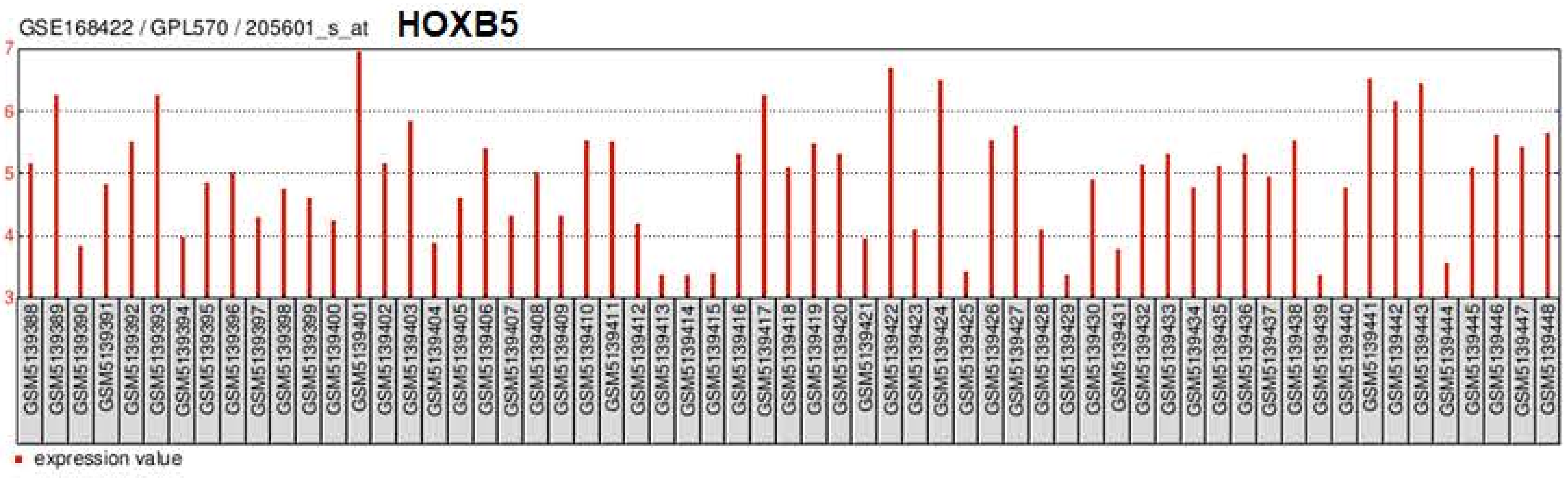

DLBCL (GSE53786)

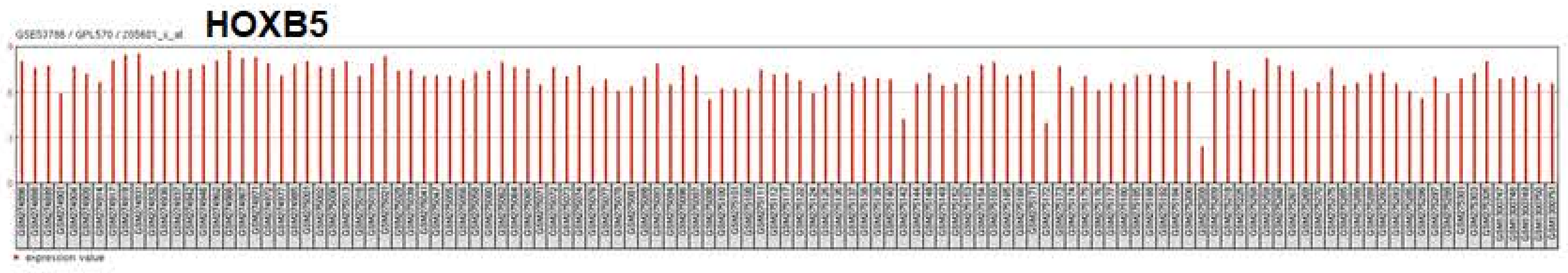

FL (GSE56311)

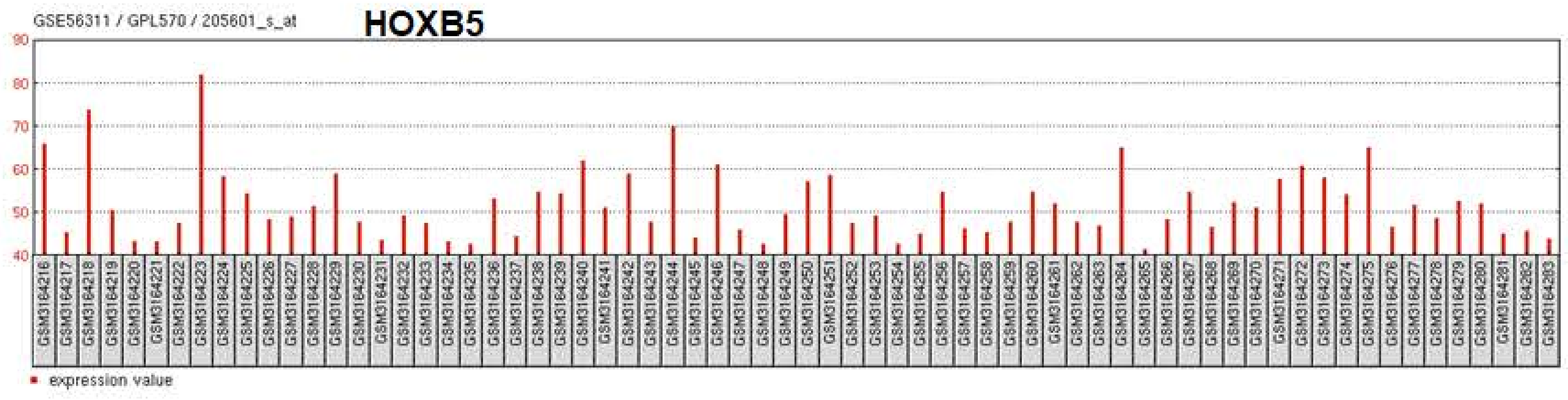

HL (GSE12453)

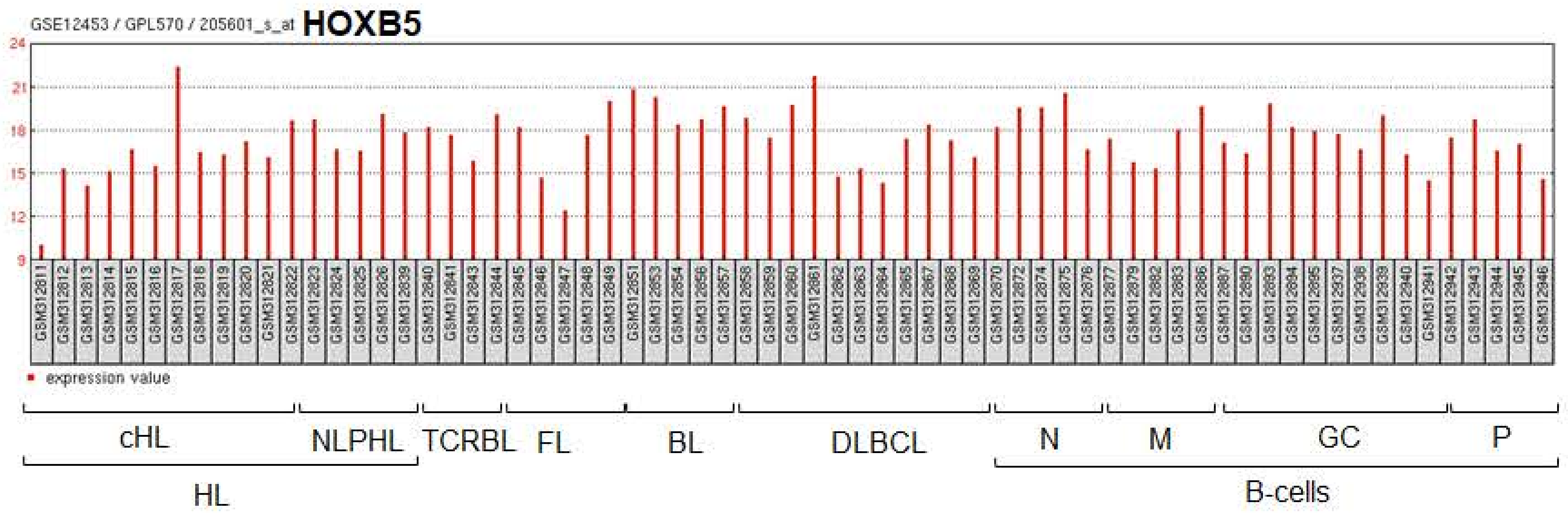

MCL, HCL, SMZL, CLL, FL (GSE16455)

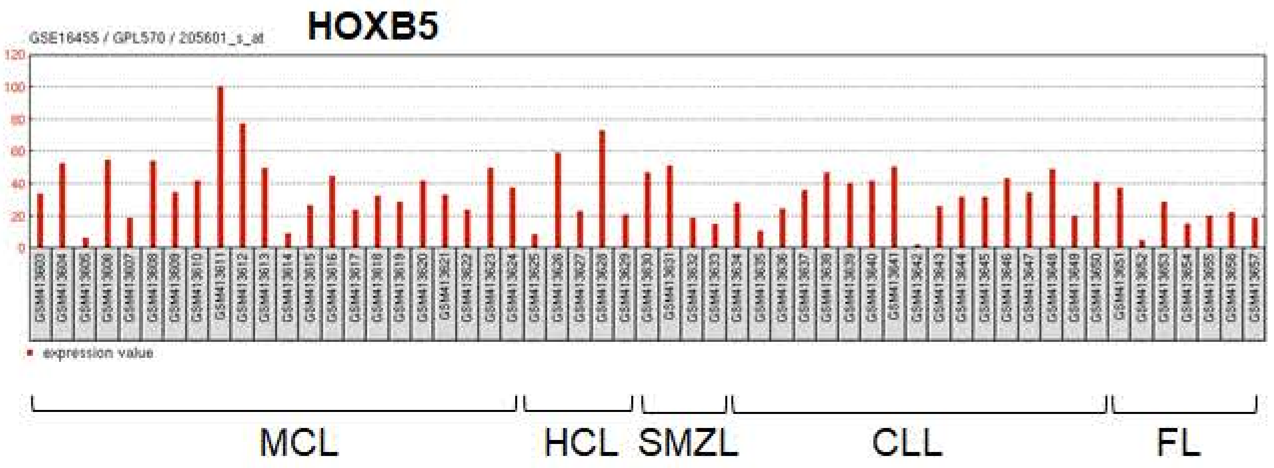

MM (GSE19554)

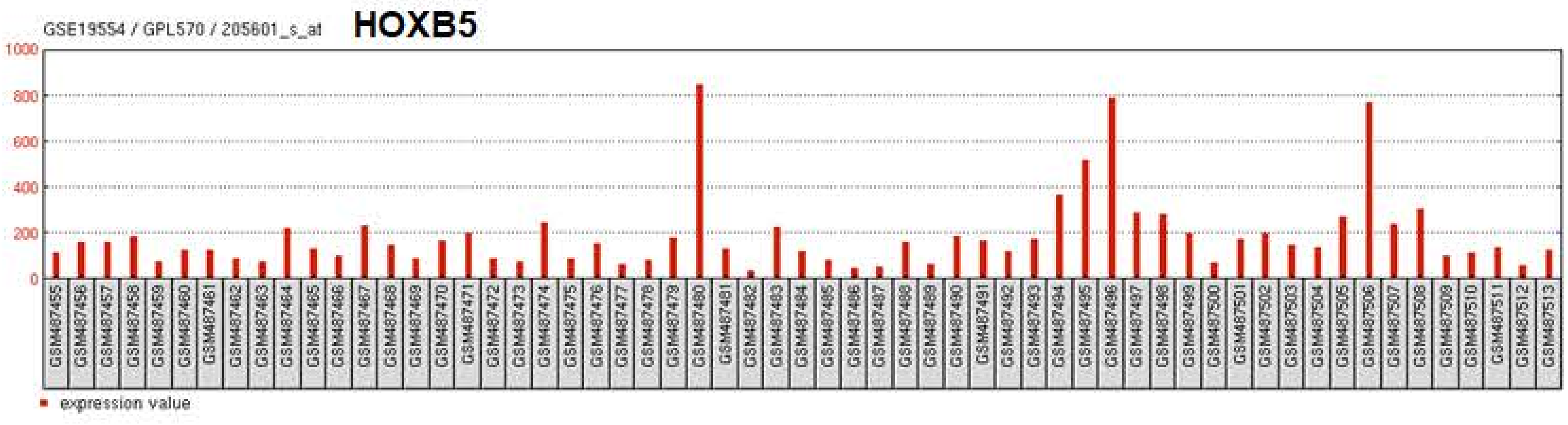

T-ALL (GSE26713)

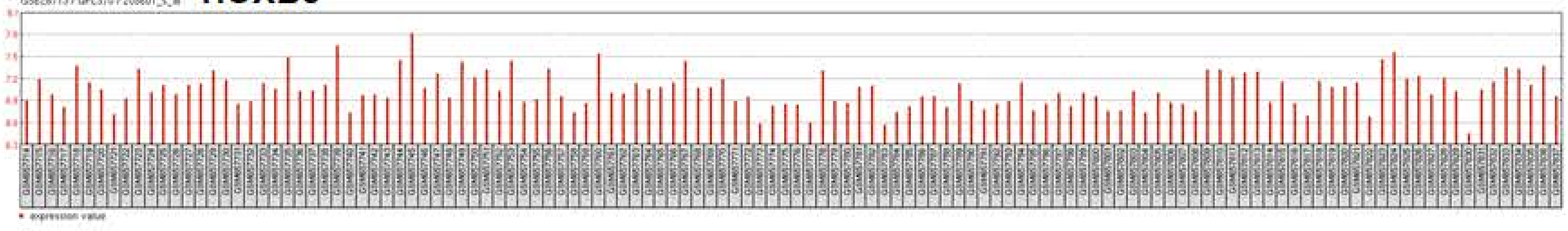

Supplementary Figure 6: Expression profiling data for HOXB genes in leukemia/lymphoma patients using public datasets.

Myelopoiesis (GSE42519)

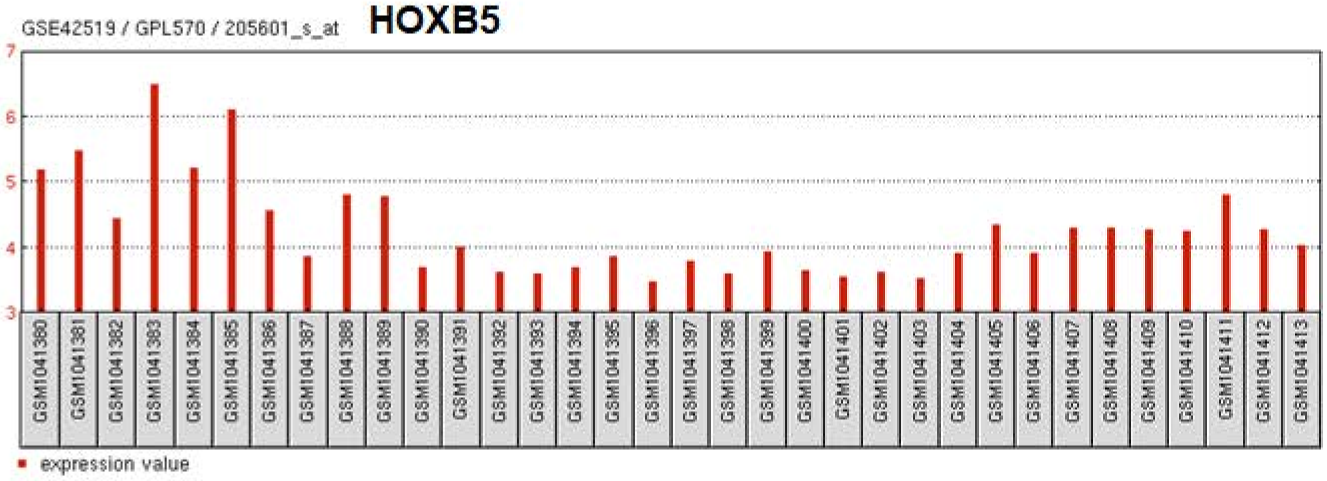

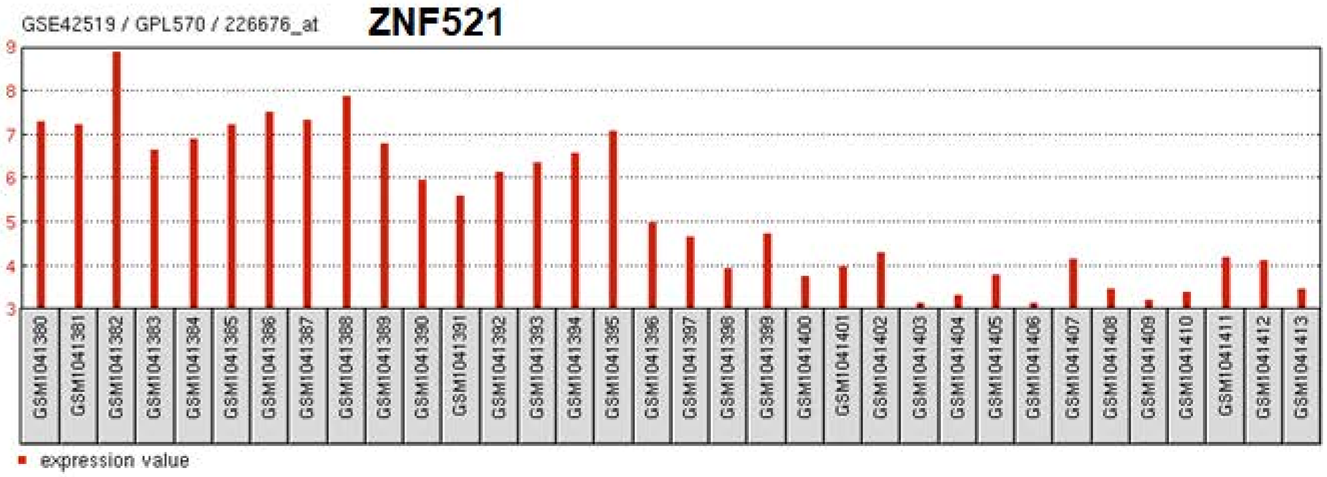

GSM1041380	HSC 1
GSM1041381	HSC 2
GSM1041382	HSC 3
GSM1041383	HSC 4
GSM1041384	MPP 1
GSM1041385	MPP 2
GSM1041386	CMP 1
GSM1041387	CMP 2
GSM1041388	CMP 3
GSM1041389	GMP 1
GSM1041390	GMP 2
GSM1041391	GMP 3
GSM1041392	GMP 4
GSM1041393	GMP 5
GSM1041394	MEP 1
GSM1041395	MEP 2
GSM1041396	early_PM 1
GSM1041397	early_PM 2
GSM1041398	early_PM 3
GSM1041399	late_PM 1
GSM1041400	late_PM 2
GSM1041401	late_PM 3
GSM1041402	MY 1
GSM1041403	MY 2
GSM1041404	MM 1
GSM1041405	MM 2
GSM1041406	MM 3
GSM1041407	BC 1
GSM1041408	BC 2
GSM1041409	BC 3
GSM1041410	BC 4
GSM1041411	PMN 1
GSM1041412	PMN 2
GSM1041413	PMN 3

Supplementary Figure 7: Expression profiling data for HOXB5 and ZNF521 in primary myelopoiesis (dataset GSE42519).

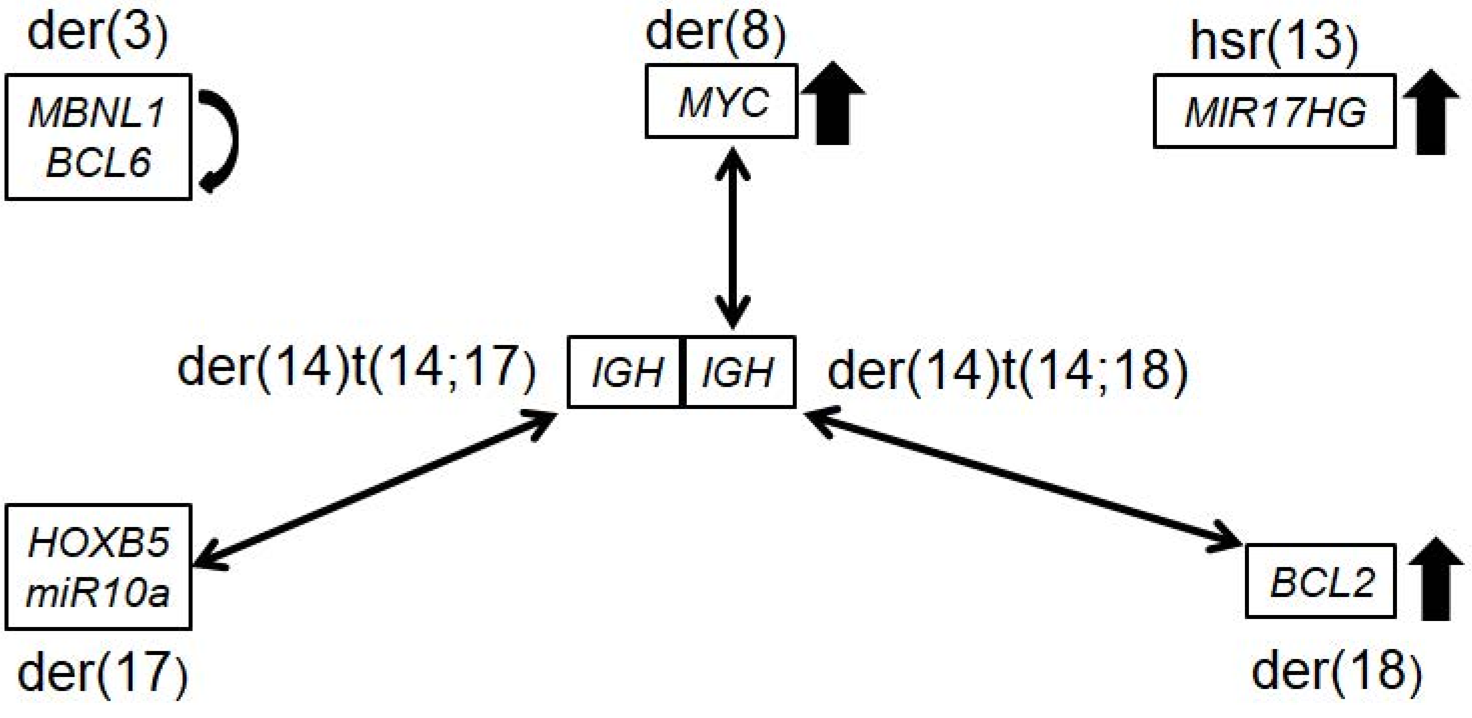

Suppl. Fig. 8: Key rearrangements present in SC-1
Diagramme shows multiple coordinated cytogenetic changes in SC-1 and the genes targeted. Note: **1)** biallelic IGH translocations targeting MYC, HOXB5, miR10a, and BCL2; **2)** genomic amplification of MYC, MIR17HG and BCL2; and **3)** juxtapositional inversion of chr.3 targeting MBNL1 and BCL6.

Supplementary Table 1

Gene ID	Gene Symbol	RC-K8	RI-1	SC-1	SU-DHL-4	SU-DHL-5	SU-DHL-6	SU-DHL-10	SU-DHL-16	controls	SC1-controls
201841_s_at	HSPB1	2,07	1,18	6,06	-4,44	-6,23	0,63	-2,63	-3,32	-1,82	7,88
213478_at	KIAA1026	-7,08	-2,74	2,98	-3,46	-5,17	-3,46	-8,12	-3,32	-4,76	7,75
236523_at	LOC285556	-3,01	-5,16	2,28	-7,83	-6,12	-8,12	-4,21	-3,32	-5,40	7,68
202718_at	IGFBP2	-1,68	-3,74	3,18	-6,38	-4,37	-5,53	-6,31	-3,32	-4,48	7,66
204971_at	CSTA	-2,70	-1,80	4,32	-2,29	-3,73	-3,29	-5,15	-3,32	-3,18	7,50
210993_s_at	SMAD1	-6,86	-2,33	3,08	-5,24	-4,42	-2,14	-6,10	-3,32	-4,35	7,43
229349_at	FLJ16517	-8,29	-2,79	2,58	-6,33	-4,21	-6,80	-1,98	-3,32	-4,82	7,40
236892_s_at	LOC404266	-7,43	-2,78	2,35	-4,74	-5,22	-4,66	-6,97	-3,32	-5,02	7,36
209278_s_at	TFPI2	-8,04	1,86	1,73	-7,00	-7,28	-7,76	-6,64	-3,32	-5,46	7,19
206163_at	MAB21L1	-6,08	-2,45	3,03	-6,04	-4,56	-1,20	-7,43	2,20	-3,65	6,69
210683_at	NRTN	-7,00	-7,12	-0,07	-8,20	-8,58	-6,51	-6,20	-3,32	-6,70	6,64
226676_at	**ZNF521**	-6,24	-5,56	1,62	-6,93	-5,84	-1,11	-6,10	-3,32	-5,01	6,63
213880_at	LGR5	-4,40	-4,57	0,67	-7,16	-4,54	-8,38	-8,97	-3,32	-5,91	6,57
239791_at	LOC404266	-3,27	-1,87	1,90	-6,70	-5,13	-6,90	-4,98	-3,32	-4,59	6,49
227798_at	SMAD1	-7,16	-3,42	2,21	-4,55	-4,32	-3,46	-3,48	-3,32	-4,24	6,45
1559394_a_at	ROR1	-5,53	0,79	1,40	-7,64	-6,43	-6,76	-5,93	-3,32	-4,98	6,37
235557_at	LOC150763	-5,13	-5,51	0,46	-6,56	-6,06	-6,43	-7,20	-3,32	-5,74	6,21
232760_at	TEX15	-6,86	-6,12	0,40	-4,53	-6,08	-8,38	-4,67	-3,32	-5,71	6,11
222921_s_at	HEY2	-3,07	-3,05	2,03	-5,47	-5,28	-2,14	-6,06	-3,32	-4,06	6,09
228280_at	MGC14289	-4,02	1,82	2,40	-3,57	-3,96	-5,09	-6,64	-3,32	-3,54	5,94
236163_at	LIX1	-3,13	-5,55	0,19	-7,64	-6,16	-8,04	-6,27	-3,32	-5,73	5,92
230487_at	LOC389440	-2,12	-2,53	3,15	-3,22	-2,16	-3,44	-2,77	-2,86	-2,73	5,88
205601_s_at	**HOXB5**	-3,41	-0,47	3,28	-2,01	-2,39	-2,05	-4,98	-2,69	-2,57	5,85
201161_s_at	CSDA	2,69	-5,30	2,48	-6,97	1,36	-5,02	-6,90	-3,32	-3,35	5,83
211796_s_at	TRBC1	-6,31	4,23	4,32	-0,91	0,47	-1,47	-3,26	-3,12	-1,48	5,80
204749_at	NAP1L3	-6,16	-4,30	0,96	-5,22	-6,54	-7,83	-4,76	1,00	-4,83	5,78
232628_at	FAM13A1	-5,16	-3,81	0,56	-4,37	-6,05	-6,27	-7,64	-3,19	-5,21	5,77
219471_at	C13orf18	-1,84	3,55	4,80	-2,74	1,24	2,42	-6,00	-3,32	-0,96	5,76
202973_x_at	FAM13A1	-2,89	-4,14	1,90	-6,29	-3,39	-5,86	-3,79	-0,48	-3,84	5,73
227792_at	---	-5,44	-5,64	0,58	-3,86	-6,42	-6,76	-4,15	-3,32	-5,09	5,67
208567_s_at	**KCNJ12**	-5,88	-1,39	1,05	-3,85	-6,18	-5,49	-6,02	-3,32	-4,59	5,64
226248_s_at	KIAA1324	-7,04	-3,73	1,17	-7,33	-4,48	-1,27	-3,71	-3,32	-4,41	5,59
203685_at	**BCL2**	2,72	5,56	5,40	-2,93	-2,53	-3,18	-4,34	3,41	-0,18	5,58
1570445_a_at	LOC441117	-7,48	-5,91	-0,27	-4,73	-6,52	-6,22	-6,67	-3,32	-5,84	5,57
230743_at	LOC404266	-4,48	-2,80	1,18	-3,59	-3,13	-6,59	-6,48	-3,32	-4,34	5,52
217728_at	S100A6	-5,97	0,58	1,53	-5,41	-0,04	-7,20	-6,51	-3,32	-3,98	5,51
207110_at	KCNJ12	-6,31	-1,74	0,96	-3,63	-5,48	-6,64	-4,41	-3,32	-4,51	5,47
200799_at	HSPA1A	-6,73	-4,87	0,57	-5,67	1,10	-6,59	-8,48	-2,98	-4,89	5,46
1553183_at	UMODL1	-8,59	-4,29	1,22	0,48	-5,36	-4,62	-3,56	-3,32	-4,18	5,40
225564_at	---	-7,43	-4,14	0,67	-6,31	-5,49	-7,20	1,15	-3,32	-4,68	5,35
205600_x_at	HOXB5	-2,59	-1,47	3,48	-1,46	-1,15	-1,63	-1,47	-3,31	-1,87	5,34
242468_at	---	-7,64	-2,40	1,04	-4,38	-5,68	-4,92	-3,14	-1,73	-4,27	5,31
1568619_s_at	LOC162073	-4,15	-3,18	0,19	-5,49	-7,38	-3,65	-8,29	-3,32	-5,07	5,26
235416_at	LOC441117	-6,67	-5,14	-0,06	-5,39	-4,84	-4,96	-6,70	-3,32	-5,29	5,23
204918_s_at	MLLT3	-0,12	-1,38	2,06	-7,48	-0,89	-2,28	-7,70	-2,34	-3,17	5,23
